# Retrospective Analysis of HPV Vaccination Attitudes and Uptake Among Medical Students: Implications for Preventive Healthcare

**DOI:** 10.3390/vaccines13121188

**Published:** 2025-11-24

**Authors:** Sylwia Kałucka, Janusz Śmigielski, Agnieszka Głowacka, Paulina Oczoś, Izabela Grzegorczyk-Karolak

**Affiliations:** 1 Department of Coordinated Care, Medical University of Lodz, 90-251 Lodz, Poland; 2 Department of Health Sciences, State University of Applied Sciences in Konin, 62-510 Konin, Poland; janusz.smigielski.stat@gmail.com; 3 Department of Nursing for Developmental Age and Health Promotion, Medical University of Lodz, 90-251 Lodz, Poland; agnieszka.glowacka@umed.lodz.pl; 4 Department of Radiological and Isotopic Diagnostics and Therapy, Medical University of Lodz, 92-213 Lodz, Poland; paulina.oczos@umed.lodz.pl; 5 Department of Biology and Pharmaceutical Botany, Medical University of Lodz, 90-151 Lodz, Poland; izabela.grzegorczyk@umed.lodz.pl

**Keywords:** attitude, HPV vaccine, nursing, medicine students, midwifery, public health, vaccination coverage, vaccine adverse events

## Abstract

Background: Human papillomavirus (HPV) vaccination remains a critical preventive strategy against HPV-related cancers, yet uptake among young adults in Poland remains suboptimal. Objectives: This study aimed to assess HPV vaccination status, determinants, and perceived barriers to vaccination among healthcare students. Methods: This retrospective survey was conducted among 1062 students of the Medical University of Lodz, including those studying Medicine, Nursing, Midwifery, and Public Health. Results: Overall, 20% of respondents reported HPV vaccination, with the highest coverage among midwifery students (26.8%) and the lowest among medical students (16.8%). The major barriers to vaccination were found to be cost and misconceptions regarding vaccination age limits. As most respondents were above 14 years old when receiving the first dose, they were not eligible for the national free vaccination program. The significant motivators were parental influence and guidance from the medical university; however, recommendations for vaccination were infrequent. Multivariable logistic regression analysis found marital status (*p* = 0.029), paternal medical education (*p* = 0.003), and prior sexual experience (*p* = 0.037) to be significantly associated with vaccination status. Adverse events were reported by 45% of vaccinated respondents, most commonly reactions at the injection site. Nursing and midwifery students more often perceived adverse events as moderate or severe, but none discontinued vaccination. Conclusions: These findings underscore the need for financial support mechanisms and targeted educational interventions to enhance HPV vaccine uptake among future healthcare professionals in Poland.

## 1. Introduction

The introduction of human papillomavirus (HPV) vaccines, initially as a quadrivalent formulation and later as a bivalent and a nonavalent formulation, has reduced the incidence of cancers associated with oncogenic HPV types in both women (cervical, anal, vaginal, vulvar, and head and neck cancers) and men (penile cancer, anal cancer, head and neck cancers: oral cavity, pharynx, larynx) [1]. Indeed, the vaccine has been found to be effective in preventing infections, genital warts and precancerous lesions of the vulva and vagina, as well as cervical precancerous lesions and cancer associated with the included HPV types [1]. Among HPV-related malignancies, cervical cancer remains the most prevalent and clinically significant form [2].

Due to its high incidence and mortality, cervical cancer continues to pose a major global health challenge. In Europe, it represents the second leading cause of cancer-related deaths among women, with approximately 33,000 new cases and 15,000 deaths reported annually, with particularly high rates observed in Eastern European countries, including Poland [3]. In Poland, an estimated 1700–2500 women die each year from cervical cancer, corresponding to five to six deaths daily [4,5].

The World Health Organization (WHO) has recommended HPV vaccination since 2010 as part of routine immunization programs. In Europe, national HPV vaccination programs were first initiated for girls in the United Kingdom in 2008, followed by gradual adoption across almost all EU/EEA countries. By 2019, 30 countries had implemented universal vaccination programs for children. Several also introduced catch-up vaccination programs for older cohorts [6,7].

In Poland, HPV vaccination has been recommended as prophylaxis against cervical cancer since 2010 [8]. However, partial reimbursement for the bivalent vaccine (Cervarix) has only been implemented for children since 2021. Completely free vaccinations under the National HPV Vaccination Programme have only been offered for boys and girls aged 11–14 since June 2023, and for those aged 9–14 since September 2024 [9]. Furthermore, at present, Cervarix is fully reimbursed for individuals up to 18 years of age, whereas only partial reimbursement is provided for older age groups. Hence, no cost-free access has been available for individuals over 18 years of age [9].

The aim of this study was to determine the HPV vaccination rates among medical students at the Medical University of Lodz across four disciplines: Nursing, Midwifery, Public Health, and Medicine. It also examined the attitudes towards HPV vaccination, taking into account that the surveyed population did not have the opportunity to benefit from the free vaccination program, which has only recently become available in Poland.

## 2. Materials and Methods

### 2.1. Participants and Study Setting

A retrospective survey was performed among the student body at the Medical University of Lodz, Poland. The target population included students of Nursing, Midwifery, Public health and Medicine. All students in the final years of their Bachelor’s (first-cycle) and Master’s (second-cycle) programs were invited to participate. For long-cycle medical studies, all students at two time points were included: the mid-degree stage, i.e., between the completion of basic education and the start of clinical classes, and the final-year stage.

The estimated total population comprised 282 Nursing students (NS), 92 Midwifery students (MS), 68 Public health students (PHS), and 939 students of Medicine (MedS). Respective minimum required sample sizes for these groups were found to be 163, 75, 58, and 273 students, as calculated using the Raosoft calculator (95% confidence level, 5% margin of error, 50% response distribution). Questionnaires were distributed and collected by members of the research team during lectures. Participation was voluntary and anonymous; all students meeting the inclusion criteria were invited to participate. In total, 1381 questionnaires were distributed, of which 1062 fully-completed forms (76.9%) were included in the analysis. Data collection was conducted from 1 March to 31 May 2025, and the required number of responses was exceeded for all groups.

### 2.2. Questionnaire Development and Validation

Data were collected using a self-administered, paper-based, anonymous questionnaire developed by the main researcher. The questionnaire was based on previous surveys conducted by the authors (earlier studies on voluntary vaccinations [10], and surveys from studies on Master’s and Bachelor’s theses) and refined through expert consultation.

The instrument was pilot-tested on 20 students from the target population to evaluate clarity and internal consistency. Feedback was used to refine wording and ensure interpretative consistency. The internal reliability of the survey was found to be high, with a Cronbach’s alpha score of 0.85. The validation procedures followed standard guidelines [11].

The final questionnaire included 21 items in two parts: the first part addressed attitudes and knowledge regarding HPV vaccination (seven single-answer questions, two multiple-choice questions, and one open-ended question), and the second part covered demographic characteristics (Table S1).

### 2.3. Data Management and Statistical Analysis

The completed questionnaires were entered into a standardized electronic database in Microsoft Excel and double-checked to ensure accuracy. The employed data entry procedures minimized transcription errors and supported subsequent statistical analysis. Only fully completed questionnaires were included in the analysis. Questionnaires with any missing responses were excluded. The dependent variable was HPV vaccination status, coded as 1 for respondents reporting at least one dose and 0 for unvaccinated respondents. Independent variables included sex, place of residence, place of work, professional experience, maternal medical education, partnership status, prior sexual experience, having children, and marital status. Categorical variables were numerically coded for statistical analysis.

Descriptive statistics were calculated. Qualitative variables are presented as absolute and relative (%) frequencies; quantitative data are presented as mean, standard deviation (SD), median, minimum, and maximum values. Associations between HPV vaccination status and independent variables were assessed using univariate and multivariate logistic regression. Chi-square tests were applied to assess associations between categorical demographic variables and vaccination status. Variables with *p* ≤ 0.05 in univariate analysis were included in multivariate models, with significance set at *p* ≤ 0.01. Analyses were performed using STATISTICA 13.1 (StatSoft Inc., Krakow, Poland).

### 2.4. Ethical Concerns

Ethical approval was obtained from the Bioethics Committee of the Medical University of Lodz (Poland) (RNN/207/23/KB) on 11 July 2023. Participation was voluntary and anonymous; returning a completed questionnaire was considered informed consent.

## 3. Results

### 3.1. Demographic Characteristics

The study population comprised 1062 respondents. Most were medical students (62.3%), followed by nursing students (23.7%), midwifery students (7.7%), and public health students (6.2%). The mean age of the participants was 22.79 ± 2.09 years, with no substantial variation between the subgroups (Table 1).

Most respondents were female (73.07%), particularly among the Midwifery (100%) and Nursing students (94.84%). Most of the remainder were male (26.76%), with the largest single group being male medical students (39.43%) (Table 1).

Most respondents were unmarried (96.05%), although over half (55.08%) reported being in a current partnership (Table 1). The majority (76.55%) had some prior sexual experience. Only 4.33% had children, with significantly higher rates observed among Nursing students (10.32%) compared to medical students (2.42%).

The vast majority of participants had no professional experience (87.48%), and among those employed, hospitals (9.89%) represented the most common workplace.

Regarding place of residence, 57.72% lived in large cities, and 22.41% in rural areas. Concerning family background, 23.16% of mothers and 9.32% of fathers of respondents had completed a medical education.

### 3.2. HPV Vaccination Status

Of the more than 1000 students included in the study, one in five had been vaccinated against HPV (Table 2).

**Figure 1:**
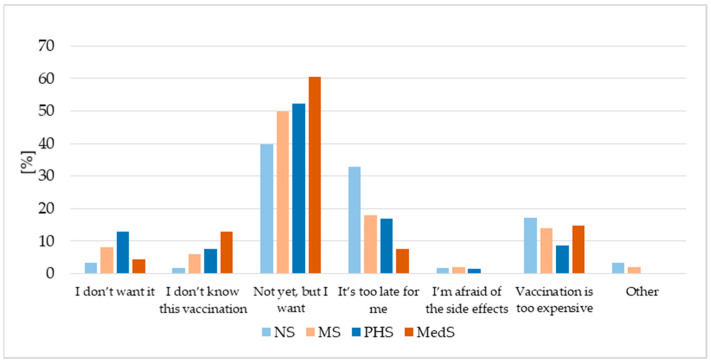
The reasons for refusal of the HPV vaccination. NS—nursing students, MS—midwifery students, MedS—medical students, PHS—public health students.

Depending on the field of study, vaccination coverage ranged from 16.77% to 26.83%, with the highest percentage observed among the Midwifery (MS) group (Table 2). Among medical students (MedS), only one in six respondents reported being vaccinated. Interestingly, the MedS group also included the highest proportion of unvaccinated students who declared an intention to be vaccinated (60.54%); in comparison, this value was less than 40% among the Nursing group (NS) (Figure 1).

Every third unvaccinated NS believed that it was already too late for them to receive an HPV vaccination. In addition, 8.72–17.24% of all respondents highlighted the cost of the vaccine as a significant barrier to vaccination (Figure 1). Depending on the field, 3.45–12.82% of participants indicated that they simply did not want to be vaccinated against HPV, and even fewer reported concerns about possible side effects (0–3.45%). These reasons are summarized in Figure 1.

### 3.3. Age at First Dose of HPV Vaccine

Most respondents received their first vaccine dose after the age of 14, and three doses were required for full vaccination (Table 3). The youngest respondent was vaccinated at the age of nine years, and the oldest at 24–25 years, suggesting that the latter group most likely initiated vaccination during their final years of study.

**Figure 2:**
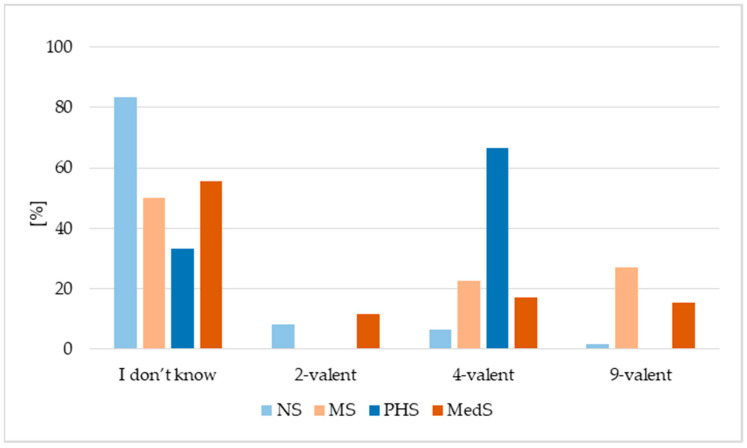
Type of vaccine used. NS—nursing students, MS—midwifery students, MedS—medical students, PHS—public health students.

The type of vaccine received was not known for the majority of the NS, MS and MedS groups (50–83.3%), but only one-third of the Public health group (PHS) (Figure 2): nearly 70% of PHS reported vaccination with the quadrivalent HPV vaccine. MS and MedS were equally likely to be vaccinated with either the quadrivalent or the nonavalent vaccine, while similar proportions of the NS group had received the quadrivalent or bivalent vaccine.

### 3.4. Information Source on HPV Vaccination

**Figure 3:**
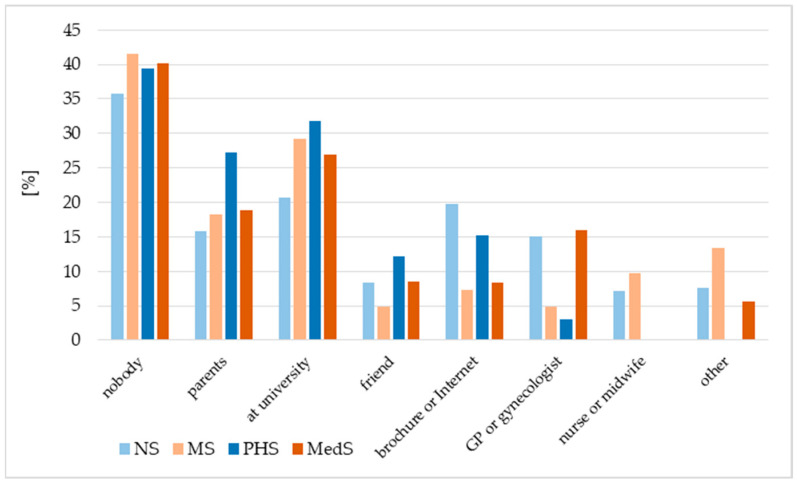
The source of recommendations about vaccination, as reported by respondents. NS—nursing students, MS—midwifery students, MedS—medical students, PHS—public health students.

The majority of students, regardless of their field of study, did not indicate any source of recommendation for HPV vaccination (Figure 3). Approximately one in five NS, one in four MedS, and one in three MS and PHS reported that the vaccine had been recommended to them at the university. A considerable proportion of students, particularly those in public health, were vaccinated on the recommendation of their parents (Figure 3).

### 3.5. HPV Vaccination Adverse Events and Future Vaccination Intentions

On average, 45% of respondents reported adverse events following vaccination. These were more common among NS (54.10%) and less common among PHS (33.33%) (Table 2).

**Figure 4:**
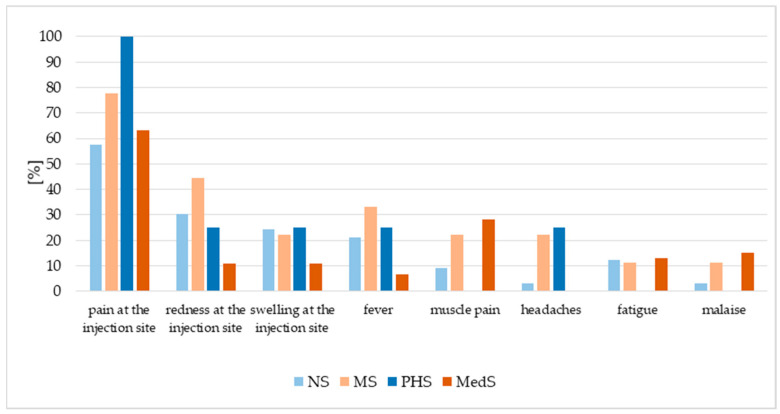
Distribution of reported adverse events after HPV vaccination. NS—nursing students, MS—midwifery students, MedS—medical students, PHS—public health students.

All PHS, 80% of MS, and more than half of the students in the other groups reported pain at the injection site (Figure 4). Other frequently-reported symptoms included redness and/or swelling at the injection site. Finally, at least one local adverse event (pain, redness, or swelling at the injection site) was reported by all PHS and MS, and by more than 70% of NS and MedS.

**Figure 5:**
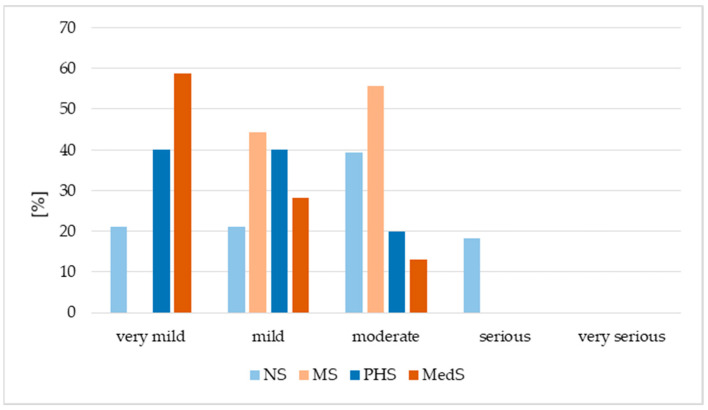
Distribution of adverse events according to severity level. NS—nursing students, MS—midwifery students, MedS—medical students, PHS—public health students.

Additionally, more than 20% of the MS group reported fever, headache or muscle pain; more than 20% of PHS noted fever or headache; more than 20% of NS reported fever, while more than 20% of MedS noted muscle pain (Figure 4). In the MedS and PHS groups, more than 80% of respondents rated the adverse events as mild or very mild, indicating low symptom severity. In contrast, over 50% of MS respondents and approximately 40% of NS respondents assessed their symptoms as moderate, while 18.18% of NS respondents reported them as severe (Figure 5).

**Figure 6:**
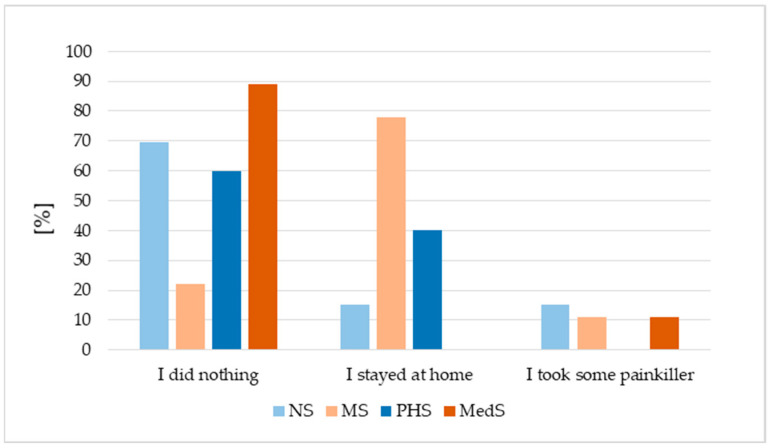
Actions taken following reported adverse events. NS—nursing students, MS—midwifery students, MedS—medical students, PHS—public health students.

Most respondents did not take any action when adverse events occurred after HPV vaccination (Figure 6). In contrast, 75% of MS reported staying at home. Importantly, no participant reported discontinuing the HPV vaccination series following adverse events (Table 2).

### 3.6. Factors Associated with HPV Vaccination Status

In the logistic regression analysis, several demographic factors were examined in relation to vaccination status (Table 4).

HPV vaccination status was most strongly associated with marital status, with married participants showing higher vaccination rates than single individuals. Moreover, prior sexual experience, having children and paternal medical education were also related to vaccination uptake. No significant association was found with place of residence, workplace or years of professional activity.

Variables that were found to be significant at *p* ≤ 0.05 in the univariate logistic regression were entered into the multivariable model (overall model *p* = 0.00026). Being married was significantly associated with higher odds of being vaccinated (adjusted OR = 2.227; 95% CI: 1.085–4.572), indicating that married respondents were approximately 2.6 times more likely to have received vaccination compared with single participants, independently of other sociodemographic characteristics included in the model. Similarly, the presence of medical education among fathers was a strong predictor of vaccination status with these respondents demonstrated almost two-times higher odds of HPV vaccination compared to others (adjusted OR = 1.887, 95% CI: 1.235–2.882), while individuals without prior sexual experience were less likely to be vaccinated compared to those with such experience (adjusted OR = 0.700, 95% CI: 0.500–0.978).

## 4. Discussion

Our findings indicate that only 20% of the studied future healthcare workers from a Polish medical university were vaccinated against HPV. This indicates a substantial gap in vaccine coverage among young adults who were too old to benefit from the nationwide HPV vaccination program for children and adolescents. In addition, the low uptake among the respondents can be attributed to inter alia initial economic barriers, limited vaccine availability at the time of introduction, and insufficient public awareness.

As of November 2024, HPV vaccination for girls had been introduced in 144 countries, accounting for about 74% of WHO member states [12]. Coverage, however, remains uneven and strongly influenced by socioeconomic and policy-related factors. In the United States, vaccination rates among adults aged 18–26 (64.2%) were markedly higher than in Poland, while Australia achieved 84.2% coverage in girls and 81.8% in boys aged 15 by 2023 [13]. Within Europe, several countries with well-established prevention programs (e.g., Sweden, Portugal, Norway, Denmark, Iceland, Malta, United Kingdom) exceeded 70% coverage, though rates across the continent still range from a dozen to over 90%, with France and Germany still reporting rates below 50% [14,15].

By June 2025, global analyses indicated that eleven countries, including Burkina Faso, Cyprus, Norway, Portugal, Turkmenistan, Uganda, and Uzbekistan, had achieved full-dose HPV vaccination coverage exceeding 90% [16]. The success of these programs has been attributed to consistent education campaigns and effective organizational frameworks. In contrast, coverage remains substantially lower in lower-middle-income countries (46.4%) compared with upper-middle-income countries (71.7%) [16]. In Asia, for instance, post-2017 implementation in Chengdu, China, increased coverage to only 24.4% [17], while uptake in Vietnamese women aged 15–29 reached merely 12% [18]. Notably, the importance of localized strategies was emphasised by data from Indonesia: coverage in a more agriculture and industry-focused area, Deli Serdang (62.09%), was found to be more than twice that of the urban district of Kota Medan (27.2%); the authors highlight the role of proactive communication and logistical preparedness [19]. Similarly, region-specific strategies in Italy, where schools were used as vaccination sites, have resulted in significantly higher coverage compared to other regions [20].

In Poland, HPV vaccination coverage was found to increase following the introduction of free vaccination in 2024; however, uptake only reached around 20% [21], i.e., considerably less than in countries that introduced fully-subsidised HPV vaccination from the start. When the vaccine was first introduced in Poland in 2007, accessibility was limited and costs were prohibitive: the cost of a single HPV vaccine dose in 2010 was approximately PLN (Polish currency; zloty) 500, with two or three doses needed for complete protection, depending on age. These expenses were high, with the mean gross monthly salary at the time being PLN 3224. Although salaries have since increased (PLN 8905 in 2024) and vaccine prices decreased (PLN 300–600 depending on the type), financial considerations can remain a barrier. Nevertheless, most unvaccinated students in the present study expressed an intention to be vaccinated.

Another potential barrier was the misconception that vaccination offers limited benefit when administered at an older age (in an age group older than that eligible for reimbursement). This perception reflects a generational knowledge gap, insofar as our respondents did not encounter extensive HPV vaccine promotion in childhood. Indeed, poor uptake is often noted among this age group, which has been attributed to poor parental engagement caused by the initial lack of reimbursement and the vaccination being promoted for girls aged 11–12 years. Recommendation has since broadened to adults of both sexes up to age 45 [22].

International comparisons confirm that national strategies can play a decisive role in increasing uptake. In Australia, the first universal HPV program for girls, later extended to boys in 2013, has resulted in marked awareness and acceptance of HPV vaccination [23]. Similarly, the United Kingdom introduced routine HPV vaccination in 2008, initially for girls, while the Netherlands implemented a programme including both boys and girls from age 10 in 2022 [24]. In the United States, the vaccine was introduced in 2006 with broad public health support ensuring access regardless of insurance status [25]; the Advisory Committee on Immunization Practices (ACIP) recommends catch-up vaccination up to age 26, and the U.S. Food and Drug Administration (FDA) has approved its use up to age 45 [26]. All these programmes resulted in increased HPV vaccine uptake.

Our findings stress the importance of increasing HPV coverage among young healthcare workers in Poland, particularly since vaccinated healthcare workers can have a positive influence on public attitudes by acting as credible advocates for immunization [27]. Evidence shows that discussions with physicians and nurses about vaccine safety significantly increase parental willingness to vaccinate their children [19,27]. However, in Poland, since the introduction of full reimbursement for children under 14 years of age, still less than 20% of eligible individuals had been vaccinated as of 2024 [14]. Hence, there is a need for targeted promotion and education initiatives led by healthcare professionals. Our present data, based on students of Medicine, Nursing, Midwifery, and Public health, indicate a wide age range (9–25 years) for the first HPV vaccine dose among students. Thus, it is recommended that the age limit for reimbursement for HPV vaccination should be extended to 26 years.

Furthermore, beyond sexual transmission, healthcare professionals may face elevated occupational risks. HPV DNA has been detected in surgical smoke during procedures such as electrosurgery, laser ablation, and cauterization of HPV-related lesions [28]. Occupational exposure has been reported in gynaecologists, dermatologists, otolaryngologists, and surgical staff working with patients with HPV [29,30]. Therefore, it is recommended that medical practitioners should be eligible for free vaccination up to the age of 45 [26].

Various social and relational factors also appear to play a role in HPV vaccine uptake, as indicated by the identification of sexual partnerships, initiation of sexual activity, and parenthood as independent determinants of HPV vaccination. The highest HPV carrier rates have been noted in women aged below 25 years, characterising the majority of respondents, which has been linked with changes in sexual partners [31]. Moreover, the lifetime risk of at least one HPV infection is estimated at 70–98% in men and 54–95% in women, depending on geography and population type [32].

Our present findings demonstrate higher vaccination rates in individuals with prior sexual experience and in married persons, possibly reflecting greater familial health awareness, preventive behaviours, or counselling received during family planning. Conversely, no association was found with sex, place of residence or professional experience, suggesting that these exert limited influence. These observations underscore the need to incorporate psychosocial determinants, particularly relationship status, into HPV vaccination strategies.

As with any vaccination, safety concerns play a key role in HPV vaccine uptake. Systematic reviews indicate the most frequent adverse events to be pain, swelling, and redness at the injection site for the bivalent vaccine, and pain and swelling for the quadrivalent vaccine [33]. In the present study, the most frequent reactions were local (viz., pain, redness, swelling), with systemic events such as fever, headache, and myalgia occurring rarely. Respondents generally rated these reactions as moderate to very mild. Comparable findings were reported in the Netherlands, where the most frequent side effects were injection-site reactions (46.5%), and headaches were noted by only a minority (8.2%) [34]. A large multicentre study spanning Europe, Asia, and the Americas also confirmed pain, redness, and swelling as the predominant side effects, with occasional headache and fatigue [35]. These adverse events are generally mild and transient, and significantly less severe than those observed following influenza or COVID-19 vaccination [36,37].

Importantly, our present findings indicate that the reported adverse events rarely required analgesics and did not require the implementation of other major actions. HPV vaccines have been extensively studied, with all three licensed types demonstrating robust safety and tolerability across age groups [33,38,39], and were confirmed as demonstrating a favourable safety profile by the Global Advisory Committee on Vaccine Safety in its 2017 position paper. After nearly two decades of international use, the evidence consistently indicates that the most common side effects are limited to mild, local post-injection reactions. This accumulated experience provides strong reassurance to healthcare professionals and the general public regarding HPV vaccine safety.

## 5. Limitations

Our study has several limitations. Initially, no exclusion criteria were applied, and the use of a paper-based questionnaire instead of an internet-based questionnaire guaranteed that the respondents belonged to the predefined study group. However, participation in the study was voluntary, which may have introduced selection bias. The student populations demonstrated a clear female predominance with women comprising over 70% of respondents, and an even higher proportion in Nursing and Midwifery. It also demonstrated a substantial imbalance in field distribution: medical students constituted the largest subgroup. These disproportions may have influenced the observed patterns. Moreover, the study did not assess the socioeconomic status of respondents or their families, as these areas are culturally sensitive among the target respondents; they may have discouraged participation or led to incomplete or unreliable responses.

Additional methodological constraints arise from the reliance on self-reported data, which inherently introduces recall bias due to potential inaccuracies in participants’ recollection of past events or behaviors. The outcomes also included subjective evaluations, creating the potential for subjective assessment bias driven by individual interpretation of survey items or personal attitudes. Furthermore, questionnaire-based research is vulnerable to respondent bias, including tendencies toward socially desirable responses or systematic misreporting. Collectively, these forms of bias may also affect the precision and internal validity of the findings [40].

## 6. Conclusions

HPV vaccination coverage among healthcare students in Poland remains insufficient, and varies slightly between health disciplines. While the population holds a generally favorable attitude toward vaccination, uptake has been hindered by misconceptions, financial barriers, and limited institutional recommendations. Importantly, adverse events are mild, and do not affect vaccination completion. The findings indicate the need for multifaceted strategies that enhance awareness, accessibility, and institutional engagement in HPV prevention, particularly within the academic environment intended to shape future healthcare professionals. Furthermore, extending reimbursement for HPV vaccination to young adults who were not eligible during childhood could effectively close the existing immunization gap and increase overall population coverage.

## Figures and Tables

**Table 1:** Socio-demographic characteristics of the respondents (n = 1062).

	NS n = 252	MS n = 82	PHS n = 66	MedS n = 662	Total n = 1062
Sex					
female	239 (94.84%)	82 (100.00%)	54 (81.82%)	401 (60.57%)	776 (73.07%)
male	11 (4.37%)	0 (0.00%)	12 (18.18%)	261 (39.43%)	284 (26.76%)
other	2 (0.79%)	0 (0.00%)	0 (0.00%)	0 (0.00%)	2 (0.19%)
Mean age	22.84 ± 2.58	22.26 ± 1.88	22.43 ± 1.42	22.88 ± 1.94	22.79 ± 2.09
Years of work in the profession					
up to 5 years	67 (26.59%)	35 (42.68%)	5 (7.58%)	18 (2.72%)	125 (11.77%)
5–10 years	0 (0.00%)	0 (0.00%)	0 (0.00%)	4 (0.60%)	4 (0.38%)
over 10 years	4 (1.59%)	0 (0.00%)	0 (0.00%)	0 (0.00%)	4 (0.38%)
I’m just learning	181 (71.83%)	47 (57.32%)	61 (92.42%)	640 (96.68%)	929 (87.48%)
Workplace					
Hospital	61 (24.21%)	29 (35.37%)	1 (1.52%)	14 (2.11%)	105 (9.89%)
Clinic	8 (3.17%)	5 (6.10%)	4 (6.06%)	4 (0.60%)	21 (1.98%)
Hospital Emergency Ward	2 (0.79%)	1 (1.22%)	0 (0.00%)	4 (0.60%)	7 (0.66%)
Not working	181 (71.83%)	47 (57.32%)	61 (92.42%)	640 (96.68%)	929 (87.48%)
Place of residence					
Village	62 (24.60%)	19 (23.17%)	13 (19.7%)	144 (21.75%)	238 (22.41%)
Small city	48 (19.05%)	11 (13.41%)	23 (34.85%)	129 (19.49%)	211 (19.87%)
Large city	142 (56.35%)	52 (63.41%)	30 (45.45%)	389 (58.76%)	613 (57.72%)
Mother with medical education					
Yes	60 (23.81%)	12 (14.63%)	11 (16.67%)	163 (24.62%)	246 (23.16%)
No	190 (75.40%)	69 (84.15%)	54 (81.82%)	495 (74.77%)	808 (76.08%)
I do not know	2 (0.79%)	1 (1.22%)	1 (1.52%)	4 (0.60%)	8 (0.75%)
Father with medical education					
Yes	10 (3.97%)	3 (3.66%)	1 (1.52%)	85 (12.84%)	99 (9.32%)
No	236 (93.65%)	78 (95.12%)	64 (96.97%)	564 (85.2%)	942 (88.70%)
I do not know	6 (2.38%)	1 (1.22%)	1 (1.52%)	13 (1.96%)	21 (1.98%)
Marital status:					
Single	239 (94.84%)	77 (93.90%)	58 (87.88%)	646 (97.58%)	1020 (96.05%)
Married	13 (5.16%)	5 (6.10%)	8 (12.12%)	16 (2.42%)	42 (3.95%)
Divorced	0 (0.00%)	0 (0.00%)	0 (0.00%)	0 (0.00%)	0 (0.00%)
Widow/widower	0 (0.00%)	0 (0.00%)	0 (0.00%)	0 (0.00%)	0 (0.00%)
Currently in a relationship					
Yes	179 (71.03%)	49 (59.79%)	44 (66.67%)	313 (47.28%)	585 (55.08%)
No	73 (28.97%)	33 (40.24%)	22 (33.33%)	349 (52.72%)	477 (44.92%)
After sexual initiation					
Yes	214 (84.92%)	61 (74.39%)	55 (83.33%)	483 (72.96%)	813 (76.55%)
No	38 (15.08%)	21 (25.61%)	11 (16.67%)	179 (27.04%)	249 (23.45%)
With children					
Yes	26 (10.32%)	4 (4.88%)	0 (0.00%)	16 (2.42%)	46 (4.33%)
No	226 (89.69%)	78 (95.12%)	66 (100%)	646 (97.58%)	1016 (95.67%)

NS—nursing students, MS—midwifery students, MedS—medical students, PHS—public health students.

**Table 2:** Comparison of HPV vaccine uptake and adverse events across four healthcare student groups.

Vaccinated	NS n = 252	MS n = 82	PHS n = 66	MedS n = 662	Total n = 1062
Yes	61 (24.21%)	22 (26.83%)	15 (22.73%)	111 (16.77%)	209 (19.68%)
No	191 (75.79%)	60 (73.17%)	51 (77.27%)	551 (83.23%)	853 (80.32%)
Adverse events	33 (54.10%)	9 (40.91%)	5 (33.33%)	46 (41.44%)	93 (44.50%)
Interruption of the series due to adverse events	0 (0.00%)	0 (0.00%)	0 (0.00%)	0 (0.00%)	0 (0.00%)

NS—nursing students, MS—midwifery students, MedS—medical students, PHS—public health students.

**Table 3:** Age at HPV vaccination.

Age	Mean ± SD	Minimum	Maximum	Median
NS	14.35 ± 2.73	12.00	25.00	13.00
MS	14.32 ± 3.46	11.00	21.00	13.50
PHS	15.07 ± 2.28	12.00	18.00	16.00
MedS	14.96 ± 3.39	9.00	24.00	14.00
Total	14.74 ± 3.16	9.00	25.00	14.00

NS—nursing students, MS—midwifery students, MedS—medical students, PHS—public health students.

**Table 4:** The logistic regression analysis for associations between demographic characteristics and vaccinated status.

Variable	Vaccinated	Univariate Logistic Regression		Multivariable Logistic Regression	
		OR (95% CI)	*p*	OR (95% CI)	*p*
Sex	female (25.13%)	Ref.			
	male (12.13%)	2.410 (0.651–8.925)	0.187		
Place of residence	village (19.41%)	Ref.			
	others (22.36%)	1.058 (0.883–1.266)	0.540		
Place of work	only learning (21.02%)	Ref.			
	working (26.28%)	1.130 (0.980–1.310)	0.100		
Years of professional experience	not working (21.02%)	Ref.			
	having experience (25.76%)	1.258 (0.883–1.792)	0.204		
Maternal medical education	no + I don’t know (20.98%)	Ref.			
	yes (24.08%)	1.240 (0.890–1.720)	0.201		
Paternal medical education	no + I don’t know (20.71%)	Ref.			
	yes (31.31%)	1.959 (1.715–2.237)	0.001	1.887 (1.235–2.882)	0.003
Partnership status	yes (21.92%)	Ref.			
	no (21.43%)	1.030 (0.780–1.360)	0.836		
Prior sexual experience	yes (20.32%)	Ref.			
	no (26.21%)	0.721 (0.517–1.004)	0.050	0.700 (0.500–0.978)	0.037
Having children	no (21.10%)	Ref.			
	yes (34.78%)	1.999 (1.186–3.367)	0.009	1.337 (0.652–2.740)	0.428
Marital status	single (20.92%)	Ref.			
	married (40.48%)	2.577 (1.364–4.854)	0.003	2.227 (1.085–4.572)	0.029

## Data Availability

The dataset we analyzed for the current study is available from the corresponding author on request.

## Supplementary Materials

The following supporting information can be downloaded at: https://www.mdpi.com/article/10.3390/vaccines13121188/s1, Table S1: The survey questionnaire.
