# Supplementary material for: Retrospective Analysis of HPV Vaccination Attitudes and Uptake Among Medical Students: Implications for Preventive Healthcare
Source: Vaccines (Basel). 2025 Nov 24;13(12):1188. doi: 10.3390/vaccines13121188 (PMC12737701; doi:10.3390/vaccines13121188)
Supplement: Supplementary file 1 [file vaccines-13-01188-s001.zip › vaccines-3970840-supplementary.pdf]

Table S1. The survey questionnaire

Question	Answers
1. Have you already been or are in the procedure of HPV vaccination	• Yes - Please transfer to question number 3<br>• No - Please, give the reason (question 2), and transfer to question number 5
2. Why, no?	• I don't want to vaccinate<br>• I don't know this vaccination<br>• Not yet, but I'm going to do it in the future<br>• It's too late for me to receive an HPV vaccine<br>• I'm afraid of the side effects<br>• The vaccination is too expensive for me.<br>• another reason ………………………………………..
3. How old were you when you received your first dose of the HPV vaccine?	…………………………………………….
4. What type of vaccine were you vaccinated with?	• I don't know<br>• bivalent<br>• quadrivalent<br>• nonavalent
5. How did you find out about HPV vaccine? (you can chose more than one answer)	• nobody<br>• my parents<br>• friends<br>• I heard about it at university<br>• my GP<br>• my gynaecologist/ urologist<br>• a nurse<br>• a midwife<br>• brochures seen at hospital/ surgery<br>• on the internet<br>• by accident<br>• others ………………………………………..
6. Had you any side effects after the vaccination?	• Yes - Complete questions 7-10<br>• No - Please transfer to question number 11
7. What side effects did you have after which ever dose of vaccine? (you can chose more than one answer)	• pain at the injection site<br>• redness at the injection site<br>• swelling at the injection site<br>• higher temperature/ fever<br>• muscle pain<br>• headache<br>• fatigue<br>• malaise<br>• diarrhoea or other digestive problems<br>• fainting<br>• others ………………………..
8. How serious were the side effects after the vaccination?	• very mild<br>• mild<br>• moderate<br>• serious<br>• very serious
9. How did you counteract the side effects?	• I did nothing<br>• I stayed at home

	• I took some painkillers<br>• I had an appointment with the GP<br>• others ....
10. Did the occurrence of side effects result in interruption of the HPV vaccination series?	• No<br>• Yes, I'm not fully vaccinated.
11. Sex	• female<br>• male<br>• other
12. Age (in years)	………………………..
13. Years of work in the profession	• up to 5 years<br>• 5-10 years<br>• over 10 years<br>• I'm just learning
14. Place of work	• hospital<br>• clinic<br>• hospital emergency ward<br>• I don't work yet.
15. Place of residence	• village<br>• small city<br>• large city
16. Does your mother have a medical education	• Yes<br>• No<br>• I don't know.
17. Does your father have a medical education	• Yes<br>• No<br>• I don't know.
18. Marital status	• single<br>• married<br>• divorced<br>• widow/widower
19. Are you in a partner relationship	• Yes<br>• No
20. Have you already initiated sexual intercourse	• Yes<br>• No
21. Do you have children?	• Yes<br>• No
